# Assessing Dynamic Cognitive Function in the Daily Lives of Youths With and Without Type 1 Diabetes: Usability Study

**DOI:** 10.2196/60275

**Published:** 2025-02-11

**Authors:** Mary Katherine Ray, Jorie Fleming, Andrew Aschenbrenner, Jason Hassenstab, Brooke Redwine, Carissa Burns, Ana Maria Arbelaez, Mary Ellen Vajravelu, Tamara Hershey

**Affiliations:** 1 Department of Psychiatry, Washington University in St. Louis, 660 S. Euclid Avenue, St. Louis, MO, 63110, United States, 1 3142738322; 2 Department of Radiology, Washington University in St. Louis, St. Louis, MO, United States; 3 Department of Neurology, Washington University in St. Louis, St. Louis, MO, United States; 4 Department of Pediatrics, Washington University in St. Louis, St. Louis, MO, United States; 5 Division of Pediatric Endocrinology, Diabetes, and Metabolism, UPMC Children's Hospital of Pittsburgh, University of Pittsburgh School of Medicine, Pittsburgh, PA, United States

**Keywords:** ecological momentary assessment, EMA, ambulatory, smartphone, continuous glucose monitoring, CGM, assessment, daily lives, youth, type 1 diabetes, diabetes, feasibility study, pilot study, glycemic control, environmental factor, phone, acceptability, young, cognitive test, app, application, mobile phone

## Abstract

**Background:**

Studies have shown a relationship between worse glycemic control and lower cognitive scores in youths with type 1 diabetes (T1D). However, most studies assess long-term glucose control (eg, years-decades) and cognition at a single time point. Understanding this relationship at a higher temporal resolution (eg, minutes-hours) and in naturalistic settings has potential clinical implications. Newer technology (eg, continuous glucose monitoring [CGM] and ecological momentary assessment) provides a unique opportunity to explore the glucose dynamics that influence dynamic cognition; that is, cognitive functions that fluctuate short-term and are influenced by environmental factors.

**Objective:**

Before we can assess this relationship, we need to determine the feasibility of measuring cognition in youths in daily life and determine the plausibility of obtaining glucose variation with CGM to be integrated with real-time cognition measures. This study’s purpose was to assess the acceptability of measuring dynamic cognition using a smartphone app and adherence to cognitive testing in daily life in youths with and without T1D. Further, we assessed CGM-derived glucose measures at temporally related timeframes to cognitive testing in naturalistic settings.

**Methods:**

Data were obtained from 3 studies including one in-laboratory study and 2 remote studies. For all studies, youths were asked to complete cognitive tests on the Ambulatory Research in Cognition (ARC) smartphone app that measured processing speed, associative memory, and working memory. For the in-laboratory study, youths completed testing 4 times during 1 session. For the remote studies, youths were asked to complete cognitive tests 5 times per day for either 10 or 14 consecutive days in daily life. Youths were asked to rate their impressions of the app. Youths with T1D wore a CGM.

**Results:**

74 youths (n=53 control; n=21 T1D) aged 4‐16 years participated. Youths generally reported liking or understanding the ARC app tasks in a laboratory and remote setting. Youths had high testing adherence in daily life (2350/3080 to 721/900, 76.3%‐80.2%) and none dropped out. The percentage of measurements within each glycemic range taken immediately before the app’s cognitive testing was 3% (28/942) low glucose, 51% (484/942) euglycemia, 23% (221/942) high glucose, and 22% (210/942) very high glucose. In the 2-hour window before each cognitive task, mean glucose was 182.5 (SD 76.2) mg/dL, SD in glucose was 27.1 mg/dL (SD 18.7), and the mean maximum difference between the highest and lowest glucose was 85.5 (SD 53.7) mg/dL.

**Conclusions:**

The results suggest that using the ARC smartphone app to assess dynamic cognitive functions in youths with and without T1D is feasible. Further, we showed CGM-derived glycemic variability at temporally associated timeframes of dynamic cognitive assessments. The next steps include using ecological momentary assessment in a fully powered study to determine the relationship between short-term glycemic control and cognition in youths with T1D.

## Introduction

Research has shown that youths with type 1 diabetes (T1D) often have slightly lower cognitive scores compared to their peers without T1D, and a relationship between worse glycemic control and lower cognitive scores [1-11]. These results highlight an important relationship between glucose and cognition in T1D. However, they are based on measures of long-term glucose control over long periods (eg, years-decades) and cognitive function tested in a laboratory setting at a single time point. Understanding this relationship at a higher temporal resolution (eg, minutes-hours) and in naturalistic settings has potential clinical implications.

Newer technology (eg, continuous glucose monitoring [CGM] and ecological momentary assessment [EMA]) provides a unique opportunity to explore the specific glucose patterns that influence dynamic cognition; that is, cognitive functions that fluctuate in the short-term and are easily influenced by environmental factors [12,13]. Using these approaches in adults with T1D, a few studies have shown an association between short-term glycemic control and cognitive functioning. Namely, studies have shown an association between significant glucose fluctuations and slower objective measures of processing speed at the moment [14], person-reported hypoglycemia and worse subjective measures of cognitive functioning later in the day [15], and increases in nocturnal hypoglycemia with slower processing speed the following day [16].

Whether this relationship or others are seen in youths with T1D is unknown. Understanding this relationship is significant given the importance of optimal cognitive functioning in academic settings in youths. However, before we can assess this relationship, we need to determine the feasibility and practicality of measuring dynamic cognition in youths in daily life. Although EMA is a feasible methodology for better understanding daily functioning in youths [17], including cognitive functioning [18], to our knowledge, no published study has assessed the feasibility of using a smartphone app to obtain EMAs of cognition in youths in the context of T1D.

This study aimed to test the feasibility of using a smartphone app called the Ambulatory Research in Cognition (ARC) app in youths. The ARC app, developed at Washington University in St. Louis, was originally designed to assess cognitive function in everyday environments in adults at risk of developing dementia [19]. Performance on ARC app cognitive tasks is sensitive to clinical status and genetic risk for Alzheimer disease [20,21] and can capture circadian fluctuations in cognition in adults at risk of Alzheimer disease [22]. The ability to capture variability in cognition in tandem with fluctuations in glucose in youths with T1D will be essential for determining the effects of glycemic variability on cognition in the daily lives of youths with T1D in future studies. Thus, in addition to testing the feasibility of the ARC app in youths, we also sought to determine the plausibility of obtaining glucose variation with CGM and integrating it with real-time measures of cognitive function using our protocol. This is important because EMA schedule selection has shown to be essential for capturing enough glycemic events throughout the day to integrate with cognitive data in adults with T1D [23].

## Methods

### Participants and Procedures

#### Recruitment

Study flyers were distributed throughout St. Louis Children’s Hospital clinics, Midwest diabetes support groups, and the Washington University Volunteers for Health Research Registry program. The flyers included a short description of this study and the research team’s contact information; interested parents were asked to contact the research team. St. Louis Children’s Hospital health care workers also provided names of clinic patients who may be interested in this study to the research team. Participants were also recruited from word of mouth approaches with enrolled families telling friends about this study. After making contact with the families, the research team conducted a phone screen meeting to determine eligibility based on the inclusion and exclusion criteria outlined in Table 1. Data were obtained from 3 separate studies in youths with and without T1D. Study 1 was conducted in the laboratory setting, and studies 2 and 3 were conducted in remote settings. Of note, the study 2 inclusion criteria included owning an iPhone (6s or newer as this was the minimum required to support the ARC app) and established use of a Dexcom G5 or G6 CGM. For study 3, we obtained additional funding that allowed us to provide iPhones to youths who did not have their own and Dexcom G6 PRO CGMs to youths who did not already use a CGM. Given that we could provide CGMs to youths with T1D, we changed this study’s protocol for study 3 from 14 days to 10 days to align with the life of a provided CGM (described in more detail below).

Figures and tables created with bioRender and REDCap (Research Electronic Data Capture) was used to support data collection [24-27].

**Table 1.** Inclusion and exclusion criteria for the pilot and feasibility studies that aimed at assessing the feasibility of using the Ambulatory Research in Cognition smartphone app to measure cognitive function in youths with and without type 1 diabetes in an in-laboratory setting (study 1), for 14 days in naturalistic settings (study 2), and for 10 days in naturalistic settings (study 3).

Study	Inclusion criteria	Exclusion criteria
Study 1: in laboratory (1 session)	Age 4‐16 years	No English fluency Unable to use a smartphone Severe mental, neurological, or medical condition that would make them unable to complete tasks Use of medications with known effects on the central nervous system (except ADHD^a^ medicines)
Study 2: real world (14 days)	Age 9‐16 years Owning an iPhone (6s or newer) Established use of Dexcom G5/G6 continuous glucose monitoring	No English fluency Unable to use a smartphone Severe mental, neurological, or medical condition that would make them unable to complete tasks Use of medications with known effects on the central nervous system (except ADHD medicines) No parent-approved screen time for participation No tablet or computer with reliable internet connection Severe phone screen cracks that would affect testing
Study 3: real world (10 days)	Age 9‐16 years	No English fluency Unable to use a smartphone Severe mental, neurological, or medical condition that would make them unable to complete tasks Use of medications with known effects on the central nervous system (except ADHD medicines) No parent-approved screen time for participation No tablet or computer with reliable internet connection Severe phone screen cracks that would affect testing

a ADHD: attention-deficit/hyperactivity disorder.

#### Study 1

Participants completed a tutorial of all the app tasks and were able to ask the research team any clarifying questions. They completed app testing 4 times with 5-minute breaks and then completed an experiential interview to assess their impressions of the app in the laboratory setting.

#### Study 2

Participants had a video call with researchers to practice with the ARC app tasks and ask the research team any clarifying questions. The research team established CGM data sharing privileges through the Dexcom Clarity app in youths with T1D. The following day, all youths were asked to complete cognitive testing in daily life 5 times per day for 14 consecutive days. They had a video call with the research team 1 and 2 weeks later to complete an experiential interview.

#### Study 3

Participants had a video call or visited the laboratory to practice the app tasks and establish CGM data sharing privileges (T1D group). The following day, all youths were asked to cognitive complete testing in daily life 5 times per day for 10 consecutive days. For both remote studies, monetary incentives were provided to encourage testing compliance as outlined in the Ethical Considerations section below.

### Ethical Considerations

The Washington University Human Research Protection Office (IRB00009237) approved all study procedures. Parents provided consent for them and their child to participate and youths provided assent. For privacy and confidentiality protection, all study data were deidentified. Compensation was provided to participants. For study 1, youths were compensated US $15 per hour (total of US $30), for their participation, paid via check. For study 2, youths could earn up to US $125 in Amazon gift cards for their participation (US $20 for participation in video calls; US $70 for being in this study for 2 weeks—US $5 per day when they completed at least 2 of the 5 cognitive task sessions; US $35 for completing tests—US $0.50 per test). For study 3, youths could earn up to US $115 in Amazon gift cards for their participation (US $25 for an introductory session via Zoom [Zoom Communications, Qumu Corporation], US $40 for an introductory session at Washington University; US $50 for being in this study for 10 days—US $5 per day if they completed at least 3 of the 5 cognitive tasks; US $25 for completing tests—US $0.50 per test). For study 2 and 3, youths who were aged ≥13 years were also given a Fitbit to track sleep and activity (data not shown) and kept the tracker as compensation.

### Measures

#### ARC App

**Figure 1.**
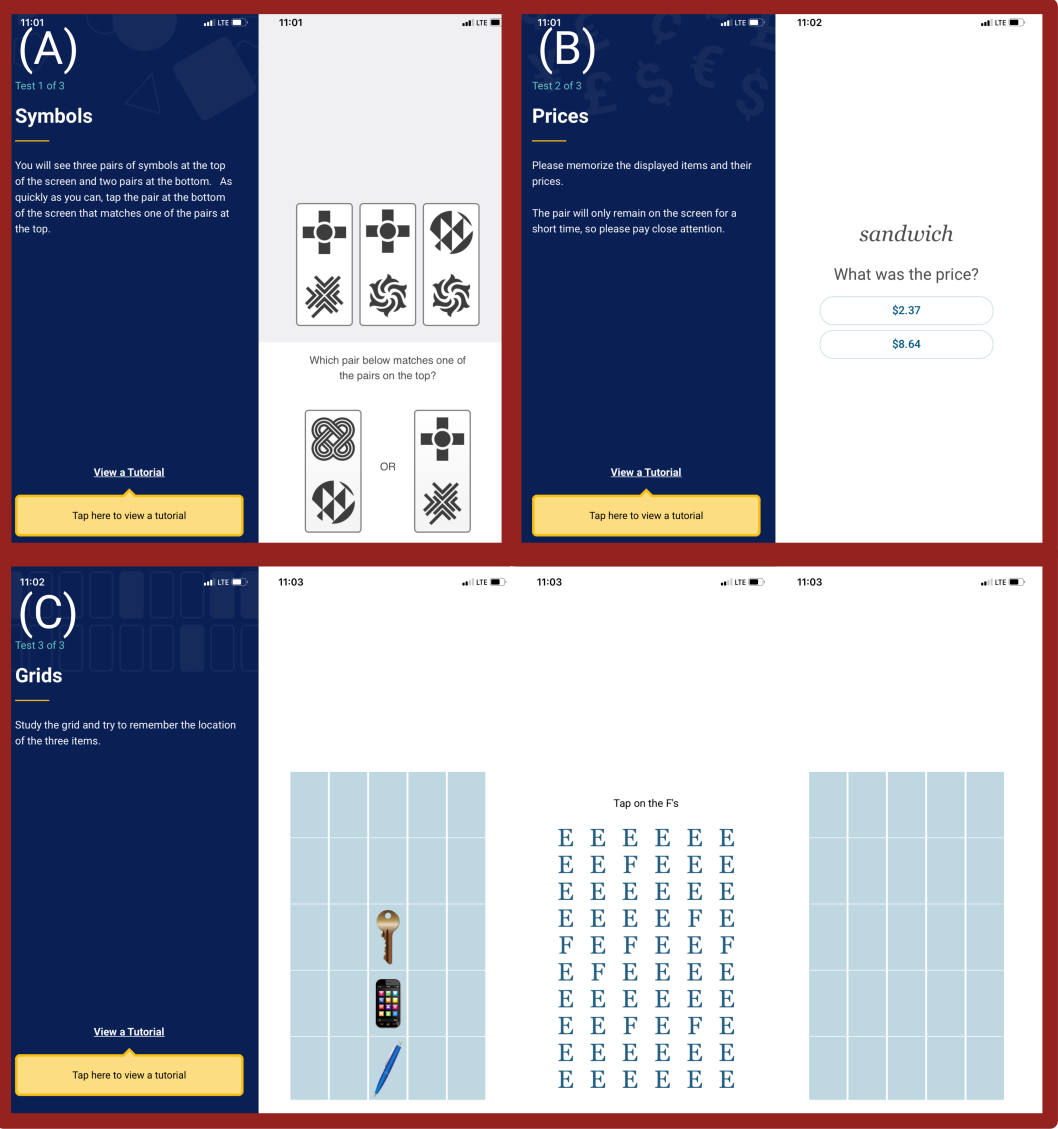
The Ambulatory Research in Cognition app being tested in this pilot and feasibility study. (**A**) The symbols task assesses processing speed. (**B**) The prices task assesses associative memory. (**C**) The grids task assesses working memory.

The ARC app is comprised of 3 tasks. The symbols task assesses processing speed by asking participants to compare visual stimuli. Participants are shown 3 cards with 2 images at the top of a screen and 2 cards with 2 images at the bottom of a screen and asked to determine which card on the bottom matches a card on the top as quickly as possible (Figure 1A). There are 12 trials. The performance score is median reaction time, calculated for trials with correct responses and reaction times above 150 ms to avoid anticipatory responses. The prices task assesses associative memory. Participants are given a household item and an associated price. They are shown 10 pairs and asked to recall the price (Figure 1B). The performance score is percent error (0%=all correct, 100%=all incorrect, and 50%=chance performance). The grids task assesses working memory. Participants are shown a grid with 3 items and asked to remember their location (Figure 1C). They are presented with a distraction task where they are shown a screen of “E”s with sporadic “F”s and asked to select the “F”s. Then they are presented with a blank grid and asked to choose where the items were located. Participants are presented with this task twice per session. The performance score is the average Euclidean distance from the selected response to the correct placement (score range: 0‐5.65; 0=perfect placement of all items and trials). There was a “tutorial” button on each task interface throughout this study so youths could reorient themselves to the tasks before completing the actual tasks being recorded for this study if needed.

Each session, including all 3 tasks, only takes about 3 minutes to complete. For both remote studies*,* youths received notifications from the app to take the tests randomly and had 2 hours to complete them. This sampling method was chosen to capture variability in cognitive function and glycemic control throughout the entire day when youths were in different contexts in daily life. Youths and their parents were able to choose the timeframes when they wanted to receive notifications and could change these times through the app to reduce the burden and reduce the likelihood of receiving a notification during times that would affect daily functioning (eg, overnight while sleeping, sleeping in on the weekend, or in class at school). Parents were not explicitly told to encourage youths to complete the sessions because the goal of using EMA methodology in this context is for youths to be able to complete testing unsupervised. Relatedly, youths were told to answer questions on the ARC app tests without help from others. All participants completed the tasks using an iPhone (6s or newer). Compliance to app testing was automatically logged electronically through an ARC app dashboard. Although the app was developed for an adult population [19], the app was used as is in our study of youths; if deemed acceptable for youths in its original state, it would be a cost-effective method that could be easily implemented into study protocols.

#### Experiential Interview

For each task in study 1 and study 2, youths were asked to rate on a scale of 1‐5 how challenging or easy the task was, how confusing or clear the instructions were, how confusing or clear the task was, and how boring or fun the task was. The Likert scale had an anchor of 3 to designate a neutral appraisal for each question. For example, “How confusing were the instructions for the symbols game?” Option 3 was “not confusing or clear.”

#### About CGM

For studies 2 and 3, participants with T1D wore a Dexcom G5/G6 CGM to measure glucose every 5 minutes. Youths who already used a Dexcom CGM changed their devices as they normally would. For study 3, if youths with T1D did not already use a Dexcom CGM, they were provided with a Dexcom G6 PRO in blinded mode to wear for the duration of this study.

### Statistical Analysis

Data were analyzed with R (R Foundation), Python (Python Software Foundation), and SPSS (IBM Corp). ARC app performance: Mean and SD were calculated for performance on each ARC app task. For study 1, Pearson correlations also assessed the relationship between age and ARC task performance (Kendall τ was used for nonnormal data), and scatterplots were created to visually appraise the age at which youths started performing better on the ARC app tasks. Further, 2-tailed *t* tests determined differences in performance between age groups. Experiential interview ratings: mean and SD were calculated for each question assessing impressions of the app tasks; a mean score greater than 3 was determined to be a positive impression of the task. ARC app adherence: Adherence was calculated as the number of tests completed divided by the number of tests offered.

CGM: Glucose variable descriptives were calculated from the raw Dexcom Clarity exports for all participants with T1D across the duration of this study, including mean, SD, percent time in range (TIR; 70‐180 mg/dL), percent time below range (<70 mg/dL), percent time high (≥180 mg/dL), and percent time very high (≥250 mg/dL). The glucose measure taken immediately before each cognitive task for each individual with T1D was extracted. Glucose obtained from the CGM were matched to the corresponding cognitive test by timestamps within each system, both set to CST. Given that glucose was assessed every 5 minutes, the closest glucose measure from CGM was taken within 5 minutes of each cognitive session. Lastly, the following glycemic variables were extracted for the 2-hour window immediately before each cognitive task for each individual with T1D: mean glucose, SD of glucose, and maximum difference in glucose (ie, highest glucose within a 2 h block minus lowest glucose within the 2 h block). Mean sensor usage during participation was calculated; Dexcom Clarity defines sensor usage as the number of days during the reporting period with at least 50% CGM readings [28]. Deletion by list was used for missing CGM data.

## Results

### Participants

**Figure 2.**
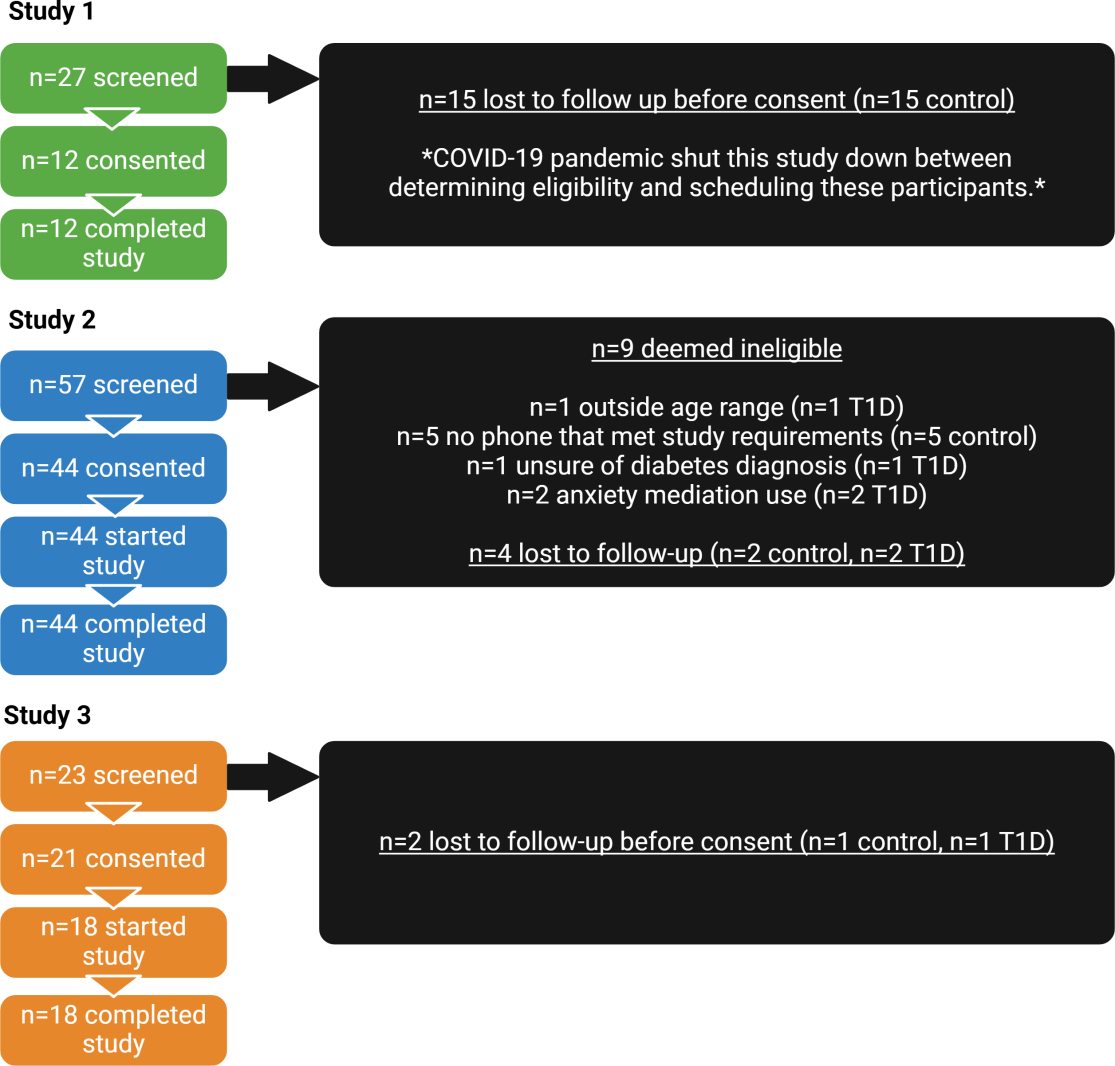
Eligibility flowchart for the pilot and feasibility studies aimed at assessing the feasibility of using the Ambulatory Research in Cognition smartphone app to assess cognitive function in the daily lives of youths with and without T1D. Study 1 was conducted in a single session in the laboratory setting in youths without T1D. Studies 2 and 3 were conducted in naturalistic settings for 14 and 10 days, respectively, in youths with and without T1D. T1D: type 1 diabetes.

Details for eligibility and consent rate are outlined in Figure 2.

Ultimately, a total of 74 participants aged 4‐16 years completed study procedures (study 1: n=12 control; study 2: n=44; n=30 control, n=14 T1D; study 3: n=18; n=11 control, n=7 T1D). No participants were voluntarily or involuntarily withdrawn from the studies. Demographic data are shown in Table 2. This study’s samples were relatively homogenous, with most participants being non-Hispanic White. For study 1, there was a glitch with the ARC app for 1 participant; thus, this participant did not have cognitive function data. For study 3, a total of 8 participants were provided with an iPhone, and all iPhones were returned to this study’s team after participation. The total annual household income for participants enrolling in this study with their own iPhone was US $131,111, and the annual household income for participants who were provided with an iPhone was US $68,750.

**Table 2.** Demographic data for the pilot and feasibility studies that aimed to assess the feasibility of using the Ambulatory Research in Cognition smartphone app to measure cognitive function in youths with and without type 1 diabetes in an in-laboratory setting (study 1), for 14 days in naturalistic settings (study 2), and for 10 days in naturalistic settings (study 3).

			Group 1	Group 2
Study 1, n			12^a^	7^b^
	Age (years), mean (SD)		10.6 (3.6)	13.1 (2.3)
	Sex, n			
		Female	7	5
		Male	5	2
	Race or ethnicity, n (%)			
		Black	3 (25)	2 (29)
		Hispanic White	1 (8)	1 (14)
		Non-Hispanic White	8 (67)	4 (57)
Study 2, n			30^c^	14^d^
	Age (years), mean (SD)		13.0 (1.7)	13.4 (2)
	Sex, n			
		Female	17	7
		Male	13	7
	Race or ethnicity, n (%)			
		Black	2 (7)	0 (0)
		Hispanic	1 (3)	0 (0)
		Non-Hispanic White	25 (83)	14 (100)
		Mixed race	2 (7)	0 (0)
	Annual household income (US $), mean (SD)		138,907 (71,860)^e^	125,231 (44,129)^f^
Study 3, n			11^c^	7^d^
	Age (years), mean (SD)		13 (2.3)	12.4 (2.2)
	Sex, n			
		Female	5	1
		Male	6	6
	Race or ethnicity, n (%)			
		Black	0 (0)	0 (0)
		Hispanic	0 (0)	0 (0)
		White	11 (100)	7 (100)
		Mixed race	0 (0)	0 (0)
	Annual household income (US $), mean (SD)		91,818 (37,031)	120,000 (47,010)^g^

a Overall

b ≥9 years old

c Control.

d Type 1 diabetes.

e n=27

f n=13

g n=6

Although the protocol for study 3 offered CGMs to use for the duration of this study for youths who did not use them, all participants enrolled already used a Dexcom CGM and used their own for this study. For study 3, one participant with T1D and his family could not sync their CGM data to Dexcom Clarity. Mean sensor usage was 97%. Average glycemic measures for youths with T1D for the entire study duration are shown in Table 3. On average, youths with T1D in our sample did not meet the recommended guidelines for percent TIR or percent time in hyperglycemia (59% vs recommended 70% TIR; 39% vs recommended 25% time in hyperglycemia) [29].

**Table 3.** Glycemic measures for youths with type 1 diabetes as measured by continuous glucose monitoring across studies 2 and 3 when youths were completing cognitive tasks on the Ambulatory Research in Cognition app in naturalistic settings for 14 and 10 days, respectively. Data are presented as mean (SD) across all days of study participation (n=20). We were unable to collect continuous glucose monitoring data in 1 participant with T1D.

Values, mean (SD)
Mean glucose (mg/dL)	176 (46)
SD glucose (mg/dL)	63.9 (17.7)
Time in range (%, 70‐180 mg/dL)	58.7 (20.6)
Time below range (%, <70 mg/dL)	2.5 (3.3)
Time high (%, ≥180 mg/dL)	38.8 (22.3)
Time very high (%, ≥250 mg/dL)	17.2 (19.1)

### Feasibility and Acceptability of Using a Smartphone App to Assess Dynamic Cognitive Function in Youths

Study 1: Youths reported liking and understanding the grids and symbols tasks but not the prices task (Table 4).

**Table 4.** Youths’ impression of and performance on the Ambulatory Research in Cognition smartphone app cognitive tasks being tested in these pilot and feasibility studies. Study 1 was conducted in the laboratory setting in youths without type 1 diabetes. Studies 2 and 3 were conducted in naturalistic settings in youths with and without type 1 diabetes for 14 or 10 consecutive days, respectively. There was an app malfunction with 1 participant from study 1, so there are only data for 11 participants.

			Symbols (processing speed), median reaction time (seconds)		Prices (associative memory), error score (%)		Grids (working memory), Euclidean distance	
			Mean (SD)	Range	Mean (SD)	Range	Mean (SD)	Range
Performance (lower score=better)								
	Study 1 (n=11)		2.4 (0.6)	1.7-4	28.6 (14.5)	6-46	0.7 (0.4)	0.1-1.7
	Study 2 (n=44)		1.8 (0.3)	1-2.7	40.6 (7.7)	10.3-50.8	0.5 (0.2)	0.1-1.3
	Study 3 (n=18)		1.9 (0.5)	1.3-2.8	40.1 (6.7)	27.1-50.8	0.5 (0.2)	0.2-0.9
Impressions								
	How hard or easy was the task? (1=very hard and 5=very easy)							
		Study 1 (n=12)	4.2 (0.7)	3‐5	2.3 (0.8)	1‐4	3.3 (1.4)	1‐5
		Study 2 week 1 (n=42)	4.3 (0.8)	2‐5	2.2 (1.1)	1‐4	4 (1)	2‐5
		Study 2 week 2 (n=43)	4.5 (0.8)	2‐5	2.6 (1.2)	1‐5	4.1 (0.9)	2‐5
	How confusing or clear were the task instructions? (1=very confusing and 5=very clear)							
		Study 1 (n=12)	4.4 (1.1)	2‐5	4.4 (1)	2‐5	4.5 (0.9)	3‐5
		Study 2 week 1 (n=42)	4.9 (0.3)	4‐5	4.8 (0.4)	4‐5	4.9 (0.4)	3‐5
		Study 2 week 2 (n=43)	4.9 (0.4)	3‐5	4.9 (0.4)	3‐5	4.9 (0.4)	3‐5
	How confusing or clear was the task itself? (1=very confusing and 5=very clear)							
		Study 1 (n=12)	4.7 (0.7)	3‐5	3.8 (1.4)	2‐5	4.3 (1.4)	1‐5
		Study 2 week 1 (n=42)	4.7 (0.7)	2‐5	4.3 (1.1)	2‐5	4.7 (0.7)	2‐5
		Study 2 week 2 (n=43)	4.8 (0.4)	4‐5	4.4 (0.9)	2‐5	4.7 (0.6)	2‐5
	How boring or fun was the task? (1=very boring and 5=very fun)							
		Study 1 (n=12)	3.6 (1)	2‐5	3.8 (0.8)	3‐5	4.5 (0.7)	3‐5
		Study 2 week 1 (n=42)	3.8 (0.9)	2‐5	2.8 (1.1)	1‐5	4.1 (0.8)	2‐5
		Study 2 week 2 (n=43)	3.8 (1)	1‐5	2.7 (1)	1‐5	4 (0.6)	3‐5

**Figure 3.**
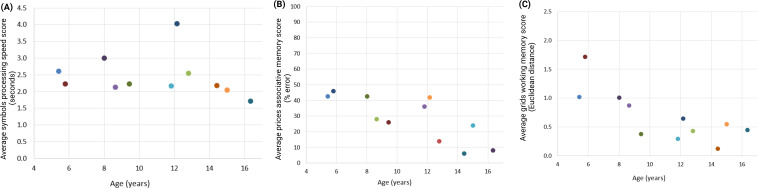
Scatterplots representing performance on each of the Ambulatory Research in Cognition smartphone app tasks and age during study 1 that aimed to test the Ambulatory Research in Cognition app in youths without type 1 diabetes at a single time point in the laboratory setting. Each dot represents an individual participant. Lower scores on each task represent better cognitive performance. The (**A**) symbols task assesses processing speed, (**B**) prices task assesses associative memory, and (**C**) grids task assesses working memory.

Performance on the tasks correlated with age (grids: *R*=−0.77, *P*=.006, n=11; prices: *R*=−0.77, *P*=.005, n=11; symbols: τ =−0.42, *P*=.07, n=11), such that younger youths performed worse than older youths. Visually, scatterplots showed that around the age of 9 years, youths started performing better (Figure 3).

**Figure 4.**
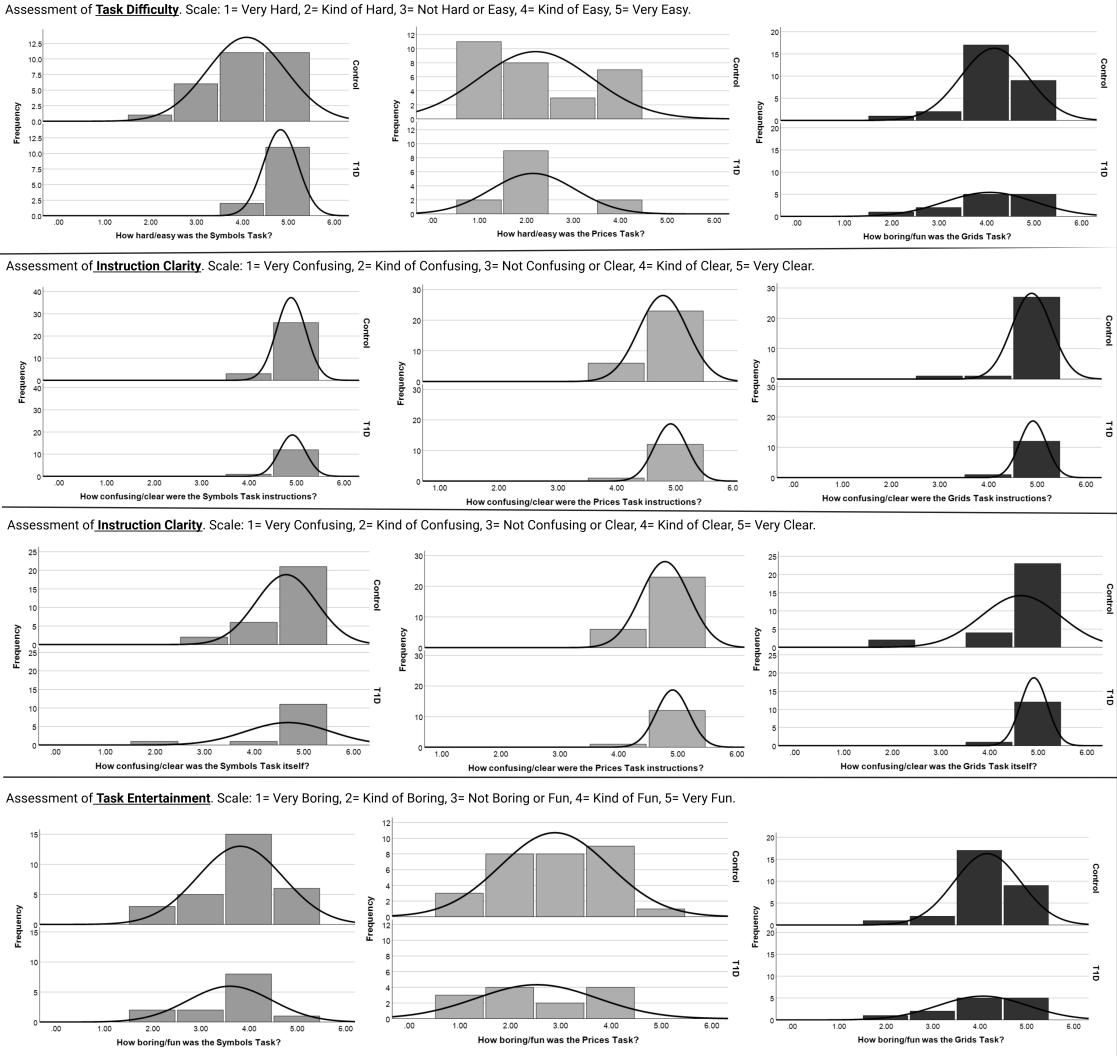
Histograms displaying ratings of the Ambulatory Research in Cognition app symbols, prices, and grids tasks from the experiential interview in study 2 which was conducted in naturalistic settings for 14 consecutive days in youths with and without type 1 diabetes. Ratings for the symbols task, which assesses processing speed, is shown in blue. Ratings for the prices task, which assesses associative memory is shown in green. Ratings for the grids task, which assesses working memory, is shown in red.

Based on these visual representations, we assessed performance in youths aged between <9 years and ≥9 years. There were statistically significant differences in performance such that youths aged <9 years had worse performance than youths aged ≥9 years on the grids task (<9 years: mean 1.2, SE 0.19; ≥9 years: mean 0.41, SE 0.10; *P*=.001) and prices task (<9 years: mean 39.8, SE 4.00; ≥9 years: mean 22.3, SE 5.19; *P*=.047). These data guided our decision to include ages 9‐16 years in study 2 and study 3. For study 2, youths completed 76.3% (SD 19) of offered tasks, and for study 3, youths completed 80.2% (SD 13) of offered tasks. Across both studies, youths reported liking and understanding the grids and symbols tasks but not the prices task (Table 4). Figure 4 shows histograms for the distribution of task rankings for the control and T1D groups.

### Glycemic Variability and Cognitive Function Data Integration

Across all participants with T1D from studies 2 and 3, the percentage of measurements within each glycemic range for the glucose measure taken immediately before cognitive testing was as follows: 3% (28/942) low glucose, 51% (484/942) euglycemia, 23% (221/942) high glucose, and 22% (210/942) very high glucose. In the 2-hour window immediately before each cognitive task, mean glucose was 182.5 (SD 76.2) mg/dL, SD in glucose was 27.1 (SD 18.7) mg/dL, and the mean maximum difference between the highest and lowest glucose was 85.5 (SD 53.7) mg/dL.

## Discussion

### Principal Findings

This study aimed to assess the feasibility of using a smartphone app to measure dynamic cognitive function in youths in daily life and to integrate this information with data obtained from CGM in youths with T1D. Our results suggest that using the ARC smartphone app to assess dynamic cognitive functions in youths with and without T1D is feasible. Further, we showed glycemic variability from CGM measures at temporally associated timeframes of dynamic cognitive function measures using our EMA schedule.

### Feasibility and Acceptability of Using a Smartphone App to Assess Dynamic Cognitive Function in Youths

Testing of the app in an in-laboratory setting revealed that youths aged younger than 9 years performed worse than youths aged ≥9 years. Differences in performance on the ARC app in the laboratory setting guided our decision to include youths aged ≥9 years in the remote testing phases of the research. Although we expected older children to perform better than younger youths simply because of normal cognitive development progression, we wanted to be cautious of including youths in remote settings who may not understand the tasks enough to be able to complete them independently. Thus, for remote testing, we chose to focus on older children whose performance was uniformly better in our in-laboratory data. Future research is needed to determine if the ARC app could be used in younger youths in remote settings, and if not, what stimuli would better engage and test cognition in younger children.

Testing the app in naturalistic settings in youths, we found that youths had high testing adherence in daily life (76%‐80%). Although we expected lower adherence in youths given the amount of time spent at school and participating in extracurricular activities, compliance in our study was similar to that of adults from a previous study using the ARC app (~80%) [19]. Further, none of all the 62 youths who started with the remote study procedures dropped out. Furthermore, youths had high appraisal for 2/3 cognitive tasks, including the grids working memory task and the symbols processing speed task. It is also important to note that providing iPhones to youths who did not have their own for participation (study 3) helped expand our reach to include youths from lower income communities compared to this study’s protocol when youths were required to have their own iPhones (study 2). Of note, all iPhones provided to participants for this study were returned to the research team which shows the logistical feasibility of this provision. Given that worse T1D outcomes have been associated with lower income [30-32], reaching youths from lower SES communities in future studies will be crucial to understand the relationship between short-term glycemic control and dynamic cognitive function. Taken together, our data support the use of the ARC smartphone app to measure dynamic cognition in youths aged 9‐16 years, with and without T1D in daily life, and highlight the logistical feasibility of providing devices to youths who do not have their own.

When integrating glucose data and cognitive function data, we found that there was variation in glucose at the time of cognitive testing and during a short timeframe immediately before cognitive testing (eg, 2 h before). Specifically, youths with T1D had dysglycemia at the time of almost half of cognitive tests (49%) and on average, experienced 86 mg/dL swings in glucose in the 2 hours immediately before cognitive testing. The ability to capture glucose at various ranges (eg, low glucose or very high glucose) and variability in glucose (eg, amount of change in glucose during short periods before cognitive testing) using this methodology will be necessary for future studies aimed at determining the relationship between short-term glycemic control and dynamic cognitive function in youths with T1D.

Of note, only 3% of cognitive tasks were completed when glucose was in the hypoglycemia range in youths with T1D. Although this small percentage does not provide a significant amount of data for assessing the effects of low glucose on cognitive function, many other glycemic variables may be important for cognitive functioning that were captured frequently in our sample, including very high glucose at the time of testing and amount of change in glucose before cognitive testing. For example, using single measures of self-monitored blood glucose in the home, Gonder-Frederick et al [33] found that severe hyperglycemia (>400 mg/dl) was associated with equally substantial deteriorations in cognition in youths with T1D than severe hypoglycemia [33]. This study highlights the need to collect measures of hyperglycemia in addition to hypoglycemia in this population. Further, the use of CGM may allow future studies to capture glycemic patterns that may predict cognitive functioning outside of standard hypoglycemia and hyperglycemia cutoffs (eg, swings in glucose).

There are study limitations to consider. First, our sample was relatively homogenous. Although we were able to reach youths from lower SES communities in study 3 when we provided iPhones for this study, our sample with T1D lacked racial and ethnic diversity. Practices are needed to better engage youths from all communities in future work. Additionally, there is a documented lag between interstitial glucose obtained from CGM and blood glucose in past literature [34,35]. Given that lag time is patient-specific [36], we chose to use the glucose measurement obtained closest to each individual cognitive test to illustrate the integration of glucose measured via CGM and cognition measured via the smartphone app; future studies assessing the relationship between short-term glucose control and dynamic cognitive function need to consider multiple glycemic features that account for lag. In the same vein, we did not ask youths to report the cause for CGM sensor errors during their participation. Thus, if youths had missing CGM data, it was unclear why (eg, intrinsic sensor failure or intentional removal of the sensor). Future EMA studies should ask participants to report the cause of missing glucose data to help account for missingness when assessing the relationship between dynamic cognitive function and short-term glucose control.

In conclusion, we found that it is feasible to obtain measures of dynamic cognitive function in youths in daily life using a smartphone app and that we could capture glucose variability for integration with measures of glucose in the daily lives of youths with T1D. Using this methodology in fully powered studies assessing the relationship between short-term glycemic control in real-time cognition would move the field toward a fuller understanding of the impacts of T1D on cognitive function in youths.

## Abbreviations

ARC: Ambulatory Research in Cognition
CGM: continuous glucose monitoring
EMA: ecological momentary assessment
REDCap: Research Electronic Data Capture
T1D: type 1 diabetes
TIR: time in range
